# A new approach for hemodynamics of varicoceles: blood flow patterns based on contrast-enhanced ultrasound

**DOI:** 10.1186/s12610-024-00249-8

**Published:** 2025-01-23

**Authors:** Penglin Zou, Gaoxiang Fan, Zheng Li, Yuchen Tao, Chao Jia, Hongmei Liang, Ruhui Tian, Qiusheng Shi, Jianlin Hu, Rong Wu

**Affiliations:** 1 Department of Ultrasound, Shanghai General Hospital, Shanghai Jiao Tong University School of Medicine, Shanghai, 200080 China; 2 Department of Reproductive Medicine, Shanghai General Hospital, Shanghai Jiao Tong University School of Medicine, Shanghai, 200080 China; 3 Department of Ultrasound, Huadong Hospital, Fudan University, Shanghai, 200040 China

**Keywords:** Male infertility, Varicocele, Contrast-enhanced ultrasound, Hemodynamics, Infertilité masculine, Varicocèle, Echographie avec Produit de Contraste, Hémodynamique

## Abstract

**Background:**

Hemodynamic alterations in the spermatic vein are implicated in infertility among patients with varicocele (VC). Contrast-enhanced ultrasound (CEUS), a powerful tool for hemodynamic analysis, remains unexplored for VC. This study aimed to demonstrate the feasibility of using CEUS to evaluate spermatic vein hemodynamics in patients with VC and establish a clear correlation between specific hemodynamic patterns and impaired semen parameters. This study included 165 patients with left-sided VC and 50 healthy volunteers. All participants underwent CEUS of the spermatic veins, along with maximum venous diameter and testicular volume measurements and serum sex hormone levels and routine semen analyses. The sperm DNA fragmentation index was measured in 146 patients with VC and 37 healthy controls.

**Results:**

The analyses revealed four distinct blood flow patterns of the spermatic vein: steady flow, intermittent stasis, intermittent reflux, and filling defect. In healthy spermatic veins, the predominant blood flow patterns included steady flow and intermittent stasis. Spermatic veins with VC exhibited a significant increase in the intermittent reflux and filling defect patterns, with the proportion rising as the clinical grade increased. The four patterns were further grouped into the “steady flow & intermittent stasis” and “intermittent reflux & filling defect” patterns for logistic regression analyses; the intermittent reflux & filling defect pattern was revealed as an independent risk factor for impaired sperm concentration, total sperm counts, progressive motility, morphology, and DNA fragmentation index.

**Conclusions:**

This study validated the feasibility of CEUS for assessing the hemodynamics of the spermatic vein and established the intermittent reflux & filling defect pattern as an independent predictor of impaired semen parameters.

**Supplementary Information:**

The online version contains supplementary material available at 10.1186/s12610-024-00249-8.

## **Résumé**

**Contexte:**

Des altérations hémodynamiques de la veine spermatique sont impliquées dans l’infertilité chez les patients porteurs de varicocèle. L’échographie avec produit de contraste (EPC), un outil puissant pour l’analyse hémodynamique, reste inexplorée pour la varicocèle. Cette étude visait à démontrer la faisabilité de l’utilisation de l’EPC pour évaluer l’hémodynamique de la veine spermatique en présence de varicocèle, et à établir une nette corrélation entre des modèles hémodynamiques spécifiques et l’altération des paramètres du sperme. Cette étude a porté sur 165 patients porteurs d’une varicocèle du côté gauche et sur 50 volontaires sains. Chez tous les participants, ont été réalisés une EPC des veines spermatiques, ainsi que des mesures du diamètre veineux maximal et du volume testiculaire, des taux sériques d’hormones sexuelles, et des analyses spermatiques de routine. L’indice de fragmentation de l’ADN des spermatozoïdes a été mesuré chez 146 des patients porteurs de varicocèle et chez 37 des témoins.

**Résultats:**

Les analyses ont révélé quatre schémas de flux sanguin distincts de la veine spermatique : flux régulier, stase intermittente, reflux intermittent et défaut de remplissage. Dans les veines spermatiques saines, les schémas de flux sanguin prédominants comprenaient un flux régulier et une stase intermittente. Les veines spermatiques avec varicocèle ont montré une augmentation significative des schémas de reflux intermittent et de défaut de remplissage, la proportion augmentant à mesure que le grade clinique augmentait. Les quatre schémas ont ensuite été regroupés en deux catégories : les modèles « flux constant et stase intermittente » et « reflux intermittent et défaut de remplissage » pour les analyses de régression logistique. Le modèle « reflux intermittent et défaut de remplissage » s’est révélé être un facteur de risque indépendant de l’altération de la concentration de spermatozoïdes, du nombre total de spermatozoïdes, de la motilité progressive et de la morphologie des spermatozoïdes, ainsi que de l’indice de fragmentation de l’ADN des spermatozoïdes.

**Conclusions:**

Cette étude a validé la faisabilité de l’échographie avec produit de contraste (EPC) pour évaluer l’hémodynamique de la veine spermatique ; et elle a mis en évidence le modèle « reflux intermittent et défaut de remplissage » comme prédicteur indépendant de l’altération des paramètres du sperme.

## Background

Varicocele (VC) is defined as an abnormal dilatation of the pampiniform plexus of veins in the spermatic cord [1–5]. VC causes venous stasis and retrograde flow, leading to several pathophysiological changes, such as scrotal hyperthermia, oxidative stress, and apoptosis. These changes contribute to ipsilateral progressive testicular atrophy, deterioration of sperm parameters, and sperm DNA damage [6–8]. Researchers have suggested that insights into the hemodynamic characteristics of the spermatic veins could aid in clinical decision-making for VC [9–11]. However, a reliable clinical method for assessing the hemodynamics of the spermatic veins is currently lacking.

Venography, a technique for accurately assessing venous hemodynamics, is considered to be the most sensitive test for VC [12, 13]. However, its invasive nature and ionizing radiation limit its use as an independent diagnostic method [14].

Ultrasound is the preferred imaging modality for VC, with Doppler ultrasound capable of detecting hemodynamic parameters such as reflux time. Numerous ultrasound-based diagnostic and grading systems have been proposed [3]. However, these systems are difficult to use, operator-dependent, obsolete, and contradictory [10]. The European Urological Association recommends basing surgical decisions on clinical staging rather than ultrasound [10], possibly due to ultrasound’s inability to reflect the pathophysiological mechanisms of VC, specifically the hemodynamics, in the same manner as venography.

Contrast-enhanced ultrasound (CEUS) is the first-line modality for evaluating vascular pathology [15, 16]. The application of CEUS has been documented in various veins [17–19], showcasing its potential in venous disease diagnostics. Although Caretta et al. [20] and Cao et al. [21] used CEUS to assess testicular microcirculation in patients with VC, no relevant research has reported the CEUS assessment of spermatic veins.

In this study, CEUS was used to evaluate the spermatic veins of healthy volunteers and patients with VC. This study aimed to explore the feasibility of this technique for mini-invasive detection of spermatic vein hemodynamics and identify new methods for the diagnosis and assessment of VC.

## Methods

### Study participants

Patients who attended the andrology clinic of Shanghai General Hospital from October 2020 to July 2024 were assessed for eligibility, including infertile men, men from infertile couples, and those seeking fertility testing because of their family planning. A total of 165 patients with left-side VC were included in the VC group. The inclusion criteria comprised left-sided VC based on physical examination following the Dubin and Amelar grading system [9]: Grade 1 is palpable only while standing during the Valsalva maneuver; Grade 2 is also palpable at rest while standing; and Grade 3 is visible through the scrotal skin. The exclusion criteria included (a) right-sided or bilateral VC, (b) previous urogenital surgery, such as orchiopexy, hydrocelectomy, or inguinal hernia repair, (c) other conditions affecting fertility, such as orchitis, epididymitis, chronic prostatitis, and vas deferens obstruction, (d) severe oligospermia, cryptozoospermia, obstructive and non-obstructive azoospermia, and multiple morphological abnormalities of the sperm flagella, and (e) poor quality of ultrasound images.

Additionally, 50 healthy volunteers who sought fertility testing in the andrology clinic were recruited as controls. The inclusion criteria for the control group were (a) the absence of VC detected during physical examination and (b) normal routine semen analysis parameters. The exclusion criteria for the control group were identical to those for the VC group.

### Ultrasound examination and image analysis

The ultrasound device used was the Aplio 900 (Toshiba, Beijing, China), equipped with a linear probe frequency of 4.0–18.2 MHz, center frequency of 12 MHz, mechanical index of 0.07, and dynamic range of 50–55 dB. The contrast agent used was SonoVue microbubbles (Bracco, Milan, Italy).

Participants were positioned supine with the perineum fully exposed at a temperature of 20–24℃. Initially, the three dimensions of the left testicle were measured using gray-scale ultrasound, and testicular volume (TV) was calculated using Lambert’s formula (TV = length × width × height × 0.71) [3]. Subsequently, the maximum venous diameter (MVD) of the left spermatic vein was measured three times, and the average value was recorded. After that, the CEUS examination was performed using the same scanning plane. The contrast agent suspension (2.4 mL in total) was injected via the antecubital vein of the left arm, followed by 5 mL of saline solution. CEUS images were recorded for 3 min. The right side was examined similarly, with a 15-min interval between the two contrast injections, which allowed for the complete elimination of the contrast agent through respiration.

CEUS images were analyzed frame-by-frame by two radiologists with over 10 years of experience in urogenital ultrasound. They evaluated the blood flow pattern of the largest spermatic vein and calculated the corresponding temporal parameters.

### Serum sex hormone levels, routine semen analysis, and DNA fragmentation index (DFI) testing

Levels of serum sex hormones, including follicle-stimulating hormone (FSH), luteinizing hormone (LH), total testosterone (T), prolactin (PRL), and estradiol (E2), were measured using an electrochemiluminescence immunoassay (COBAS 6000; Roche Diagnostics GmbH, Basel, Switzerland).

Routine semen analysis was performed according to the fifth edition of the World Health Organization Laboratory Manual for the Examination and Processing of Human Semen [22]. The Kruger criteria were used for morphological analysis, and the staining technique used was Diff-Quick. Normal semen parameters were defined based on the reference values provided in the manual: sperm concentration ≥ 15 × 10^6^/mL, total sperm counts ≥ 39 × 10^6^/mL, progressive motility ≥ 32%, and normal morphology ≥ 4%. Parameters below the reference values were considered impaired.

The DFI test was performed on 146 participants in the VC group and 37 participants in the control group, as the other participants declined to undergo this examination. A sperm chromatin structure assay was used for DFI testing. The reference values for the DFI in our laboratory are: ≤ 15% indicates good DNA integrity, 15–30% indicates general DNA integrity, and ≥ 30% indicates poor DNA integrity. In this study, a DFI > 15% was defined as impaired DNA integrity.

### Statistical analysis

Continuous variables are expressed as means ± standard deviations, and categorical variables are expressed as frequencies (percentages). The unweighted kappa statistic was used to evaluate the agreement between observers in the blood flow pattern. Intra-class correlation (ICC) under a two-way random model with absolute agreement was employed to evaluate the agreement of the observers in CEUS quantitative parameters. One-way analysis of variance was utilized to compare differences between quantitative data, and post-hoc multiple pairwise comparisons were performed using the Bonferroni test. Categorical variables were compared using the chi-square test. Univariate and multivariate analyses were performed using binary logistic regression. Statistical analyses were performed using SPSS version 25.0 (IBM, Armonk, NY, USA), and *p* < 0.05 was considered statistically significant.

## Results

### Baseline characteristics of the study population

**Fig. 1:**
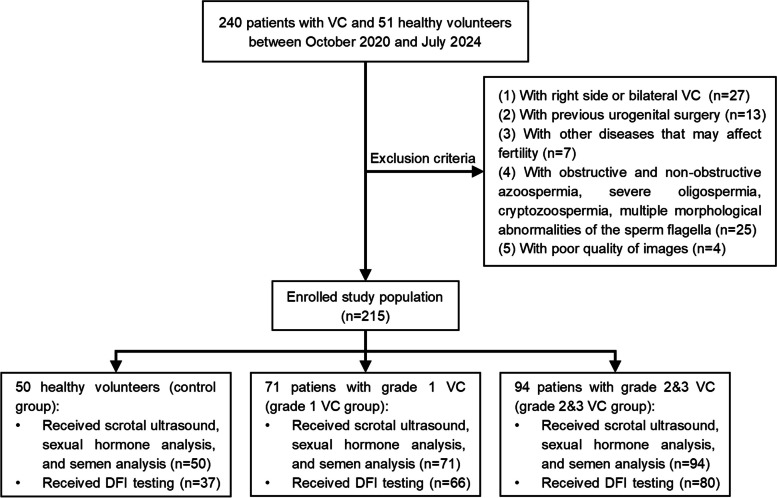
Flow chart of this study. VC Varicocele, DFI DNA fragmentation index

In this study, 50 healthy volunteers and 165 patients were enrolled (Fig. 1). Among the patients, 71 were categorized into the VC grade 1 group, whereas 94 were categorized into the VC grades 2 & 3 group. The baseline characteristics of the three groups are presented in Table 1. There were significant differences in the left MVD, total sperm counts, progressive motility, and DFI among the three groups (all *p* < 0.05). As the clinical grade increased, the left MVD (1.8 ± 0.4 mm, 2.5 ± 0.2 mm, and 3.2 ± 0.6 mm, respectively) and DFI (14.9 ± 6.0%, 19.7 ± 12.7%, and 21.5 ± 13.1%, respectively) showed a gradual increase. The total sperm counts (263.6 ± 219.0 × 10^6^, 199.0 ± 143.7 × 10^6^, and 166.2 ± 143.1 × 10^6^, respectively) and progressive motility (50.0 ± 11.4%, 44.0 ± 16.1%, and 40.5 ± 15.4%, respectively) gradually decreased.

**Table 1:** The baseline characteristics of the study population

	Control group	VC group		*p*
		Grade 1	Grade 2&3	
Number	50	71	94	–
Age (years)	30.5 ± 4.9 (21–44)	32.1 ± 4.2 (24–43)	31.7 ± 5.8 (18–57)	0.219
BMI (kg/m^2^)	24.5 ± 4.3 (18.0–32.7)	25.4 ± 3.6 (18.3–32.3)	25.7 ± 3.6 (18.3–36.1)	0.156
Gray-scale ultrasound parameters				
Left MVD (mm)	1.8 ± 0.4 (0.8–2.2)	2.5 ± 0.2 (2.0–2.9)	3.2 ± 0.6 (2.4–5.4)	< 0.001
Right MVD (mm)	1.6 ± 0.4 (0.8–2.5)	1.5 ± 0.4 (0.7–2.2)	1.5 ± 0.3 (0.8–2.2)	0.081
Left TV (mL)	15.2 ± 4.4 (8.4–27.7)	13.5 ± 4.3 (8.2–29.0)	14.0 ± 3.9 (8.2–23.6)	0.065
Right TV (mL)	16.2 ± 4.0 (9.2–24.5)	14.5 ± 4.4 (8.0–29.9)	15.6 ± 4.2 (8.1–25.1)	0.084
Reproductive hormone level				
FSH (mIU/mL)	4.4 ± 1.6 (1.5–9.1)	5.2 ± 3.0 (1.3–17.1)	4.5 ± 1.9 (1.9–13.2)	0.077
LH (mIU/mL)	4.3 ± 1.5 (2.2–8.7)	4.3 ± 1.8 (1.6–8.1)	4.4 ± 1.8 (1.3–10.5)	0.909
T (ng/mL)	5.2 ± 2.4 (2.5–12.4)	5.0 ± 2.6 (1.4–14.6)	5.3 ± 2.5 (1.2–14.3)	0.667
PRL (ng/mL)	12.0 ± 6.7 (1.1–45.1)	11.6 ± 9.1 (2.1–47.7)	11.9 ± 9.4 (2.1–75.8)	0.964
E2 (Pmol/L)	148.3 ± 82.6 (58.5–368.7)	130.1 ± 65.4 (39.4–304.8)	148.2 ± 88.0 (34.7–384.7)	0.297
Semen parameters				
Volume (mL)	3.0 ± 1.1 (0.7–6.0)	2.8 ± 1.1 (0.8–7.0)	2.7 ± 1.1 (0.2–7.0)	0.174
Concentration (10^6^/mL)	80.2 ± 51.8 (16.3–219.2)	72.4 ± 45.8 (8.0–216.0)	62.2 ± 45.3 (7.0–214.1)	0.080
Total sperm counts (10^6^)	263.6 ± 219.0 (45.0–824.0)	199.0 ± 143.7 (14.0–618.0)	166.2 ± 143.1 (10.8–748.4)	0.004
Progressive motility (%)	50.0 ± 11.4 (32.0–86.0)	44.0 ± 16.1 (11.0–75.1)	40.5 ± 15.4 (13.0–70.0)	0.002
Normal morphology (%)	7.8 ± 3.0 (4.0–16.0)	7.4 ± 4.4 (2.0–25.0)	6.7 ± 4.1 (1.0–21.0)	0.233
DFI (%)^a^	14.9 ± 6.0 (4.4–24.7)	19.7 ± 12.7 (2.2–54.2)	21.5 ± 13.1 (3.4–67.0)	0.022

The table shows the comparison of baseline characteristics between the control and VC group

Continuous variables are presented as medians means ± standard deviations (ranges) and compared using one-way analysis of variance

*p* < 0.05 was considered statistically significant

*VC* Varicocele, *BMI* Body mass index, *MVD* Maximum venous diameter, *TV* Testicular volume, *FSH* Follicle-stimulating hormone, *LH* Luteinizing hormone, *T* Total testosterone, *PRL* Prolactin, *E2* Estradiol, *DFI DNA* Fragmentation index

^a^The number of DFI tests performed in the three groups was 37, 66, and 80, respectively

**Fig. 2:**
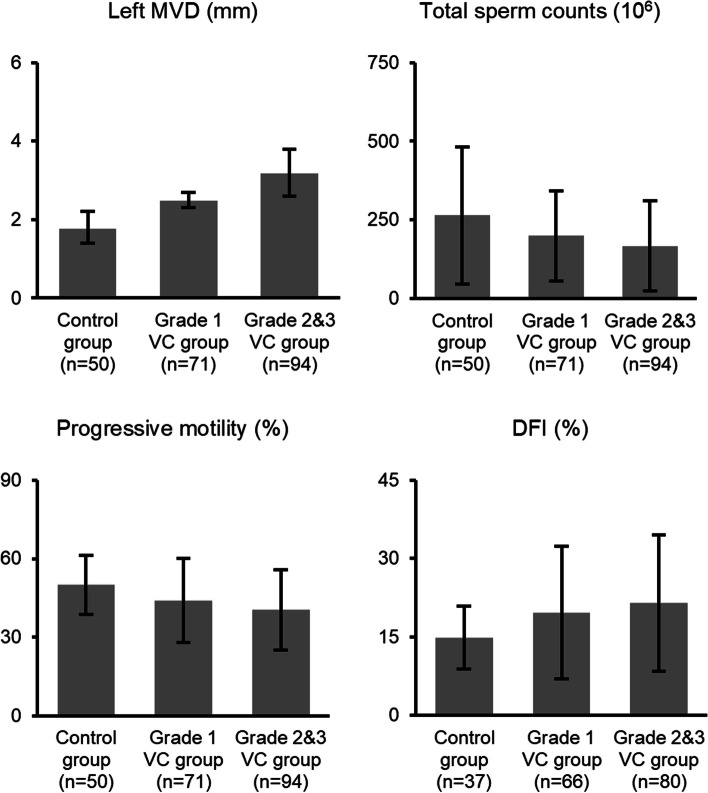
Bonferroni’s post-hoc test for the left MVD, total sperm counts, progressive motility, and DFI. MVD maximum venous diameter, VC varicocele, DFI DNA fragmentation index

Post-hoc multiple pairwise comparisons are depicted in Fig. 2. The left MVDs of all three groups were statistically different in pairwise comparisons (all *p* < 0.001). The total sperm counts, progressive motility, and DFI of the VC grades 2 & 3 group were significantly higher than those of the control group (all *p* < 0.05), and there were no significant differences in other pairwise comparisons (all *p* > 0.05).

### CEUS characteristics of the spermatic veins

**Fig. 3:**
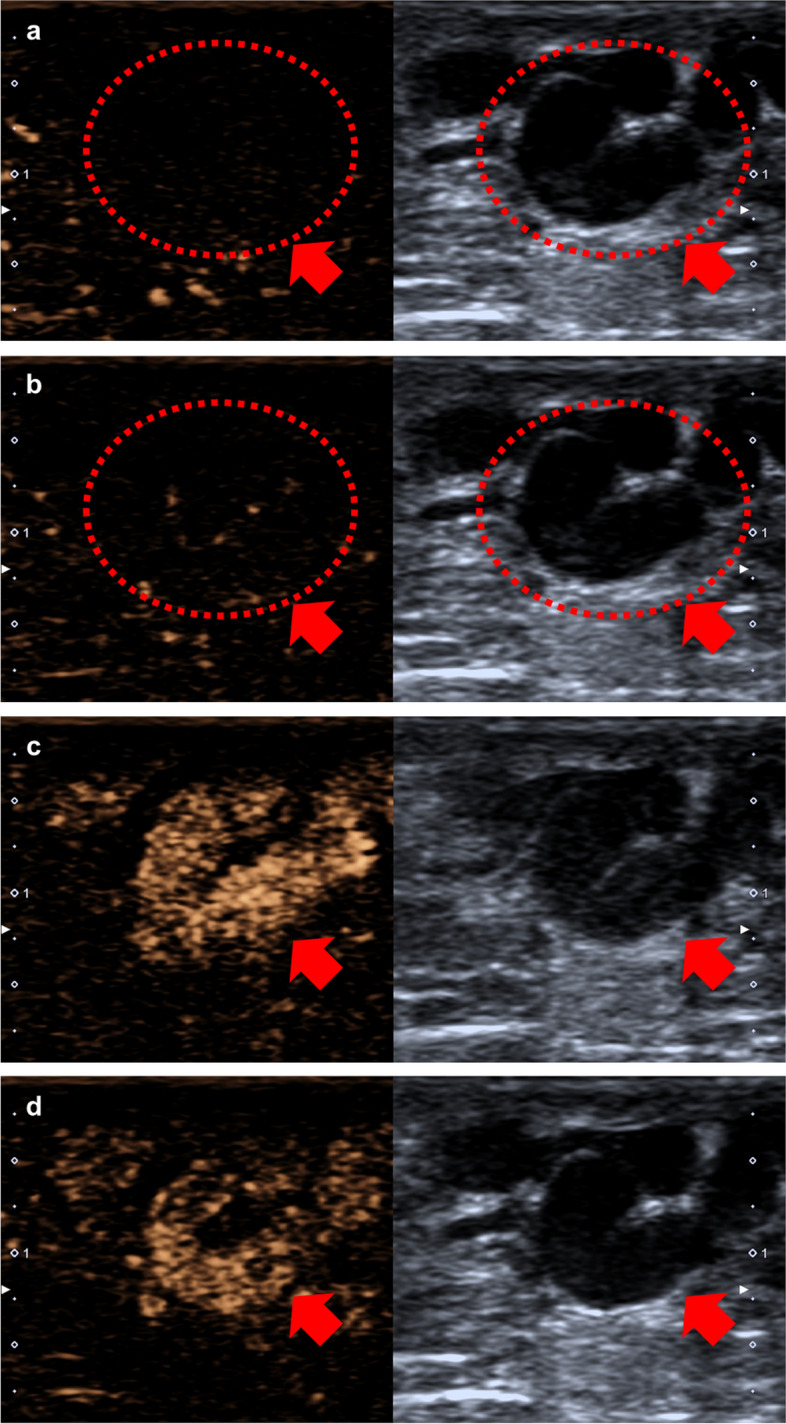
Example of CEUS images. **a** No microbubbles appeared in the spermatic vein before contrast injection. **b** Microbubbles began to enter the spermatic vein after contrast injection. The number of microbubbles entering the spermatic vein gradually increased to the peak (**c**), then gradually decreased (**d**), and finally became zero. The red circles and red arrows indicate the spermatic vein. CEUS contrast-enhanced ultrasound

In this study, CEUS was performed on the bilateral spermatic veins of 215 participants, and all were completed safely with no significant adverse events. CEUS images are shown in Fig. 3.

The movement direction of microbubbles in the spermatic vein was divided into three types: forward, reflux and pause. It is defined as:

1. Forward: The direction of microbubbles entering the spermatic vein is defined as forward, which corresponds to the normal direction of testicular blood returning to the heart.
2. Reflux: The direction opposite to the forward direction is defined as reflux.
3. Pause: When analyzing the CEUS images frame-by-frame, a lack of obvious microbubble displacement in at least two frames is defined as a pause.

Based on the changes in the movement direction of microbubbles in the largest spermatic vein, the blood flow patterns can be classified into the following four types:

1. Steady flow: The microbubbles move forward steadily without obvious pauses or reflux. An additional movie file shows this in more detail (see Additional file 1).
2. Intermittent stasis: The microbubbles move forward with intermittent brief pauses. (see Additional file 2).
3. Intermittent reflux: The microbubbles move forward with intermittent brief episodes of reflux before returning to the forward direction (see Additional file 3).
4. Filling defect: No contrast agent is observed entering the spermatic vein during the examination (see Additional file 4).

For the blood flow patterns of the control group, VC grade 1, and VC grades 2 & 3 groups, the kappa coefficients of the two observers were 0.809, 0.819, and 0.839, respectively, indicating almost perfect agreement. For the intermittent stasis pattern, the pause times assessed by the two observers were 1.3 ± 0.5 s (range, 0.4–2.1 s) and 1.4 ± 0.4 s (range, 0.4–2.7 s), respectively, with an ICC value of 0.387, indicating poor agreement. For the intermittent reflux pattern, the reflux times assessed by the two observers were 1.2 ± 0.6 s (range, 0.4–2.1 s) and 1.3 ± 0.5 s (range, 0.4–2.5 s), respectively, with an ICC value of 0.634, indicating moderate agreement.

### Distribution of blood flow patterns

**Fig. 4:**
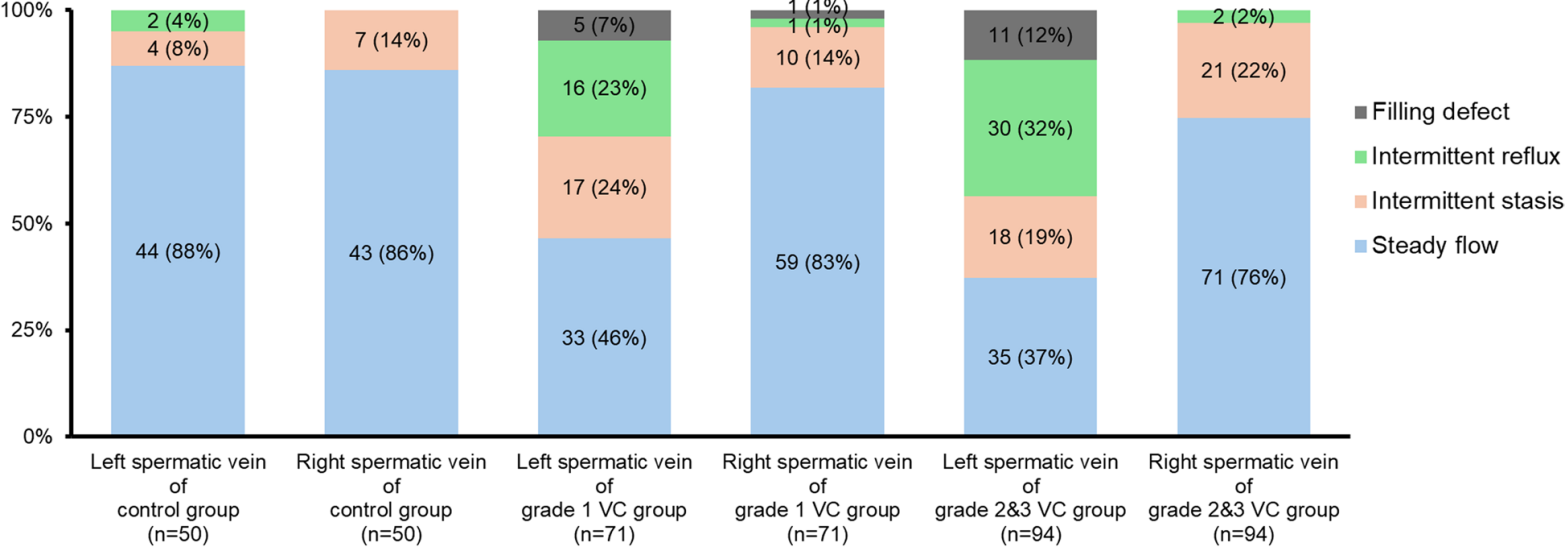
Distribution of blood flow patterns in the study population

The distribution of blood flow patterns in the bilateral spermatic veins of the study population is illustrated in Fig. 4. The chi-square test results showed no significant differences in the distribution of blood flow patterns of the spermatic veins without VC, including the bilateral sides of the control group, the right side of the VC grade 1 group, and the right side of the VC grade 2 & 3 group. However, pairwise comparison with the left side of the VC grade 1 group and the left side of the VC grades 2 & 3 group showed significant differences (both *p* < 0.05). The proportion of cases with intermittent reflux pattern was greater in the spermatic veins with higher clinical grades than in those with low clinical grades (VC grades 2 & 3: 32% vs. VC grade 1: 23% vs. no VC: 0–4%), and the proportion of cases with filling defect pattern was greater than in those with low clinical grade (VC grades 2 & 3: 12% vs. VC grade 1: 7% vs. no VC: 0–1%).

### Baseline characteristics of patients with VC with different blood flow patterns

Based on the blood flow pattern of the left spermatic vein, the VC group was divided into four subgroups with baseline characteristics presented in Table 2. A significant difference was observed in progressive motility among the four groups (*p* < 0.001). There was no significant difference in the progressive motility between the steady flow and intermittent stasis groups, the intermittent reflux and filling defect groups (both *p* > 0.05), and the rest of the pairwise comparisons were statistically different (all *p* < 0.05).

**Table 2:** The baseline characteristics of VC groups with different blood flow patterns of the left spermatic vein

	Steady flow	Intermittent stasis	Intermittent reflux	Filling defect	*p*
Number	68	35	46	16	–
Age (years)	31.1 ± 4.0 (21–41)	32.0 ± 5.4 (18–44)	33.0 ± 6.4 (24–57)	31.6 ± 5.1 (22–41)	0.293
BMI (kg/m^2^)	26.0 ± 3.3 (18.3–32.3)	25.7 ± 4.1 (18.5–36.1)	25.3 ± 3.7 (18.3–32.2)	24.2 ± 3.5 (19.9–32.2)	0.265
Gray-scale ultrasound parameters					
Left MVD (mm)	2.8 ± 0.6 (2.3–5.4)	2.9 ± 0.5 (2.3–4.7)	2.9 ± 0.6 (2.0–4.8)	2.9 ± 0.5 (2.4–4.3)	0.737
Right MVD (mm)	1.4 ± 0.4 (0.7–2.2)	1.5 ± 0.4 (0.9–2.2)	1.4 ± 0.3 (0.8–2.2)	1.6 ± 0.4 (1.0–2.2)	0.129
Left TV (mL)	14.0 ± 4.3 (8.2–29.0)	13.5 ± 3.9 (8.3–22.2)	13.1 ± 3.7 (8.2–23.6)	15.4 ± 3.9 (9.1–23.2)	0.250
Right TV (mL)	15.5 ± 4.6 (8.1–29.9)	14.4 ± 3.9 (8.2–22.0)	14.7 ± 4.1 (8.0–24.6)	16.7 ± 4.0 (12.7–25.0)	0.261
Reproductive hormone level					
FSH (mIU/mL)	5.1 ± 2.9 (1.6–17.1)	4.6 ± 1.9 (1.5–9.3)	4.7 ± 2.4 (1.3–13.2)	4.4 ± 1.7 (1.8–7.7)	0.661
LH (mIU/mL)	4.5 ± 2.0 (1.3–9.9)	4.2 ± 1.8 (2.3–10.5)	4.4 ± 1.6 (1.9–8.1)	4.0 ± 1.4 (2.1–7.2)	0.755
T (ng/mL)	5.1 ± 2.6 (1.6–14.3)	5.2 ± 2.6 (1.4–12.3)	5.3 ± 2.6 (2.0–14.6)	4.9 ± 2.5 (1.2–11.0)	0.919
PRL (ng/mL)	12.5 ± 11.1 (2.1–75.8)	13.4 ± 10.0 (2.8–43.6)	9.6 ± 5.8 (2.1–27.2)	11.9 ± 6.0 (2.9–25.6)	0.271
E2 (Pmol/L)	133.8 ± 76.1 (34.7–321.5)	143.8 ± 78.7 (44.6–371.6)	156.6 ± 86.2 (48.6–384.7)	115.0 ± 70.3 (48.2–248.3)	0.253
Semen parameters					
Volume (mL)	2.7 ± 1.1 (1.1–7.0)	2.5 ± 0.9 (0.8–4.2)	2.9 ± 1.4 (0.2–7.0)	2.7 ± 0.9 (1.3–4.4)	0.492
Concentration (10^6^/mL)	72.6 ± 45.6 (10.0–216.0)	67.1 ± 48.8 (8.0–209.1)	57.2 ± 44.8 (7.0–213.5)	67.0 ± 40.4 (9.0–149.8)	0.377
Total sperm counts (10^6^)	190.7 ± 131.1 (15.4–642.3)	170.4 ± 147.5 (20.0–748.4)	165.1 ± 158.0 (10.8–566.4)	201.3 ± 152.9 (11.7–485.8)	0.714
Progressive motility (%)	48.8 ± 12.0 (20.0–74.5)	45.2 ± 14.7 (15.0–75.1)	33.1 ± 16.1 (11.0–65.3)	31.8 ± 14.2 (13.0–61.3)	< 0.001
Normal morphology (%)	7.6 ± 4.0 (3.0–25.0)	7.9 ± 4.8 (2.0–21.0)	5.7 ± 3.8 (1.0–18.0)	6.2 ± 4.5 (2.0–20.0)	0.059
DFI (%)^a^	18.7 ± 12.0 (2.6–48.1)	20.4 ± 15.3 (2.2–67.0)	23.8 ± 13.4 (3.4–52.6)	20.7 ± 7.8 (5.7–40.9)	0.288

The table shows the comparison of baseline characteristics between VC groups with different blood flow patterns of the left spermatic vein

Continuous variables are presented as medians means ± standard deviations (ranges) and compared using one-way analysis of variance

*p* < 0.05 was considered statistically significant

*VC* Varicocele, *BMI* Body mass index, *MVD* Maximum venous diameter, *TV* Testicular volume, *FSH* Follicle-stimulating hormone, *LH* Luteinizing hormone, *T* Total testosterone, *PRL* Prolactin, *E2* Estradiol, *DFI DNA* Fragmentation index

^a^The number of DFI tests performed in the four groups was 58, 32, 41, and 15, respectively

### Association of blood flow patterns with impaired semen parameters

Based on the results stated above, the blood flow patterns of the left spermatic vein were dichotomized into “steady flow & intermittent stasis” and “intermittent reflux & filling defect,” and logistic regression analysis was performed with other baseline parameters to identify risk factors for impaired semen parameters. Other baseline parameters included age, body mass index (BMI), left MVD, left TV, FSH, LH, T, PRL, and E2.

Univariate analysis revealed that the intermittent reflux & filling defect pattern was a risk factor for impaired sperm concentration, total sperm counts, progressive motility, morphology, and DFI (all *p* < 0.05, Table 3). The sensitivity of the intermittent reflux & filling defect pattern in predicting impaired sperm concentration was 66.7%, specificity was 64.7%, negative predictive value (NPV) was 96.1%, and positive predictive value (PPV) was 12.9%. The sensitivity of the intermittent reflux & filling defect pattern in predicting impaired total sperm counts was 73.3%, the specificity was 66.0%, the NPV was 96.1%, and the PPV was 17.7%. The sensitivity of the intermittent reflux & filling defect pattern in predicting impaired progressive motility was 79.5%, the specificity was 77.7%, the NPV was 91.3%, and the PPV was 56.5%. The sensitivity of the intermittent reflux & filling defect pattern in predicting impaired morphology was 72.4%, the specificity was 69.9%, the NPV was 92.2%, and the PPV was 33.9%. The sensitivity of the intermittent reflux & filling defect pattern in predicting an impaired DFI was 47.1%, the specificity was 74.6%, the NPV was 48.9%, and the PPV was 73.2%. Among the baseline parameters, the left MVD was statistically correlated with impaired total sperm counts (OR = 2.246, 95% CI = 1.046–4.820, *p* = 0.038).

**Table 3:** Binary logistic regression analysis to confirm reflux and filling defect patterns as independent risk factors for impaired semen parameters (*n* = 165)

	Without adjustment		With adjustment^a^	
	OR (95% CI)	*p*	OR (95% CI)	*p*
Impaired sperm concentration	3.667 (1.056–12.737)	0.041	3.998 (1.059–15.095)	0.041
Impaired total sperm counts	5.338 (1.619–17.604)	0.006	5.443 (1.493–19.840)	0.010
Impaired progressive motility	13.539 (5.796–31.626)	< 0.001	14.694 (5.910–36.534)	< 0.001
Impaired morphology	6.082 (2.491–14.854)	< 0.001	7.807 (2.906–20.975)	< 0.001
Impaired DFI^b^	2.614 (1.271–5.380)	0.009	2.893 (1.336–6.266)	0.007

The table shows the results of binary logistic regression analysis to confirm reflux and filling defect patterns as independent risk factors for impaired semen parameters

*OR* Odds ratio, *CI* Confidence interval, *DFI DNA* fragmentation index, *BMI* Body mass index, *MVD* Maximum venous diameter, *TV* Testicular volume, *FSH* Follicle-stimulating hormone, *LH* Luteinizing hormone, *T* Total testosterone, *PRL* Prolactin, *E2* Estradiol

*p* < 0.05 was considered statistically significant

^a^ Adjusted for age, BMI, left MVD, left TV, FSH, LH, T, PRL, and E2

^b^ The number of DFI tests performed was 146

Multivariate analyses were performed to estimate the predictive value of the intermittent reflux & filling defect pattern for impaired semen parameters. After adjusting for all the above baseline factors, the association of the intermittent reflux & filling defect pattern with impaired sperm concentration, impaired total sperm counts, impaired progressive motility, impaired morphology, and an impaired DFI was sustained (Table 3). The associations between impaired semen parameters with baseline parameters were not statistically significant (data not shown).

## Discussion

This study introduces a novel, mini-invasive approach to assess the hemodynamics of spermatic veins. We identified four distinct blood flow patterns of the spermatic vein: steady flow, intermittent stasis, intermittent reflux, and filling defect. Healthy spermatic veins predominantly exhibit steady flow and intermittent stasis patterns, and the other two patterns are more prevalent in spermatic veins with VC. Additionally, after further categorization for logistic regression analyses, the intermittent reflux & filling defect pattern was revealed as an independent predictor of impaired semen parameters.

Dysfunction of the spermatic vein is a significant contributor to VC-induced semen parameters impairment. Exploring the hemodynamics of VC is crucial to improving the clinical diagnosis and treatment of VC [9–11]. However, it is challenging to study the hemodynamics of spermatic veins because of the complex and diverse structure of the pampiniform plexus. This study offers CEUS as a new method to assess the hemodynamics of the spermatic vein. The principle of this method is similar to that of venography [12, 13], as it records and analyzes the spatiotemporal characteristics of the contrast agent in the blood vessel to obtain hemodynamic information. The CEUS technique utilizes microbubbles — small, inert gas-filled spheres encapsulated in a phospholipid shell with a size of 2–8 μm — to enhance image clarity and contrast. Microbubbles possess several key characteristics [15]. The safety profile of microbubbles is well established, with no evidence of cardiac, hepatic, thyroid, or renal toxicity, and no obvious adverse reactions occurred during this study. Microbubbles can oscillate non-linearly in the diagnostic ultrasound field, generate harmonic frequencies, and produce stable and clear contrast images. Additionally, since microbubbles are similar in size to red blood cells, they remain strictly intravascular and do not pass through the vascular endothelium, unlike contrast media in CT and MRI, which may affect image quality. Finally, microbubbles have good blood traceability, meaning their dynamic characteristics align closely with blood flow. These advantages ensure the safety and accuracy of CEUS, making it the first-line modality for evaluating vascular lesions [15–19].

CEUS offers unique advantages over venography and Doppler ultrasound, which are classical methods for detecting hemodynamics. CEUS is mini-invasive and does not produce ionizing radiation, facilitating a greater possibility of clinical application than venography. Although venography has been used to study the blood flow pattern of VC [12, 13], it only shows the blood reflux from the testicular vein to the renal vein at a macroscopic scale. CEUS, on the other hand, provides detailed information on the blood flow in the spermatic vein around the testis and epididymis, which is theoretically more directly related to testicular function. Doppler ultrasound is currently the first-line method to detect the blood flow of the spermatic vein. The commonly used parameters are reflux time [23], maximum reflux velocity [24], and reflux pattern [9–11] during the Valsalva maneuver. However, owing to challenges in fully calibrating the incidence angle of ultrasound and variations in the participants’ understanding and execution of the Valsalva maneuver, Doppler ultrasound systems are associated with operational difficulties, significant inter-operator differences, and contradictory results [10]. CEUS can make up for the inherent limitations of Doppler ultrasound, including a lower signal-to-noise ratio, lower sensitivity for slow flow, and technical artifacts, thus significantly improving blood flow visualization [15].

Four blood flow patterns of the spermatic vein were summarized in this study. The flow of blood in the spermatic vein, including advancement, pause, and reflux, is mainly affected by the pressure gradient and venous valve function [25]. The venous pressure of the normal spermatic vein is higher than that of the left renal vein, allowing blood to return to the left renal vein when blood flow exhibits a steady flow pattern. Peripheral venous pressure fluctuates within the normal range as influenced by physiological activities such as heartbeat and respiration [26]. When the peripheral venous pressure is at a high value, the left renal vein pressure exceeds the spermatic vein pressure, and the blood in the spermatic vein tends to reflux. If the valve is functioning normally or is minimally damaged, it can block reflux and pause blood flow, at which point blood flow exhibits an intermittent stasis pattern. If the degree of valve function damage is high and the blood breaks through the valve and backflows, the blood flow pattern shows intermittent reflux. After a brief pause or reflux, the left renal vein pressure falls back below the spermatic vein pressure, and blood resumes an antegrade flow. A small portion of testicular blood returns via the cremasteric and differential veins, which have small anastomotic branches with the spermatic vein and are normally not opened [11]. When the anastomotic branch opens pathologically, blood will enter the cremasteric and differential veins through the anastomotic branch. Currently, although the spermatic vein proximal to the anastomotic branch is markedly dilated, no contrast agent can be observed, resulting in a filling defect pattern. The above pathophysiological mechanisms may explain the distribution of blood flow patterns in this study: intermittent reflux and filling defect patterns were almost exclusively found in the diseased spermatic vein, and the proportion increased with an increase in the clinical grade.

In this study, we analyzed the correlation between the blood flow pattern of the left spermatic vein and semen parameters in the VC group. The progressive motility of the intermittent reflux group (33.1 ± 16.1%) and filling defect group (31.8 ± 14.2%) was significantly lower than that of the steady flow group (48.8 ± 12.0%) and intermittent stasis group (45.2 ± 14.7%), but there were no significant differences in semen parameters between the steady flow group and intermittent stasis group or between the intermittent reflux group and filling defect group. Theoretically, the lesion severity in the filling defect group was higher than that in the intermittent reflux group [11], but the difference in semen parameters between the two groups was not significant, which may be attributable to the small sample size of this study. For this reason, logistic regression analysis was performed after dichotomizing the blood flow patterns. The intermittent reflux & filling defect pattern was an independent risk factor for impaired semen parameters with high sensitivity (66.7%, 73.3%, 79.5%, and 72.4%, respectively) and specificity (64.7%, 66.0%, 77.7%, and 69.9%, respectively) in predicting impaired sperm concentration, total sperm counts, progressive motility, and morphology. Conversely, it had high specificity (74.6%) but low sensitivity (47.1%) in predicting an impaired DFI. These findings are consistent with the pathophysiological mechanisms of spermatogenic dysfunction previously described [4–6].

### Limitations of the study

This study has the following major limitations. First, Due to the small sample size, we combined VC grades 2 and 3 and simplified the blood flow pattern into a binary category in the logistic regression analysis. A larger sample size in future studies could address this shortcoming. Second, this study was performed in the lying position and without the Valsalva maneuver because the subjectivity of the Valsalva maneuver and the body sway caused by the standing position can reduce accuracy. Moreover, Cavallini et al. [10] found that continuous hemodynamic abnormalities can affect spermatogenesis, whereas temporary reflux (mainly during the Valsalva maneuver) has little effect on testicular function. Thus, hemodynamics during quiet breathing may be more relevant to spermatogenesis. Third, further analysis of quantitative parameters was not performed due to significant inter-observer variability in reflux and pause times. The current clinical application of CEUS mainly relies on subjective and qualitative evaluation and interpretation by the physician performing the examination [15]; thus, the results of this study still have potential value for clinical application. Finally, although no apparent adverse events occurred during this study, we did not assess the potential long-term effects of CEUS on the testis and other tissues. To the best of our knowledge, there are no literature reports on long-term adverse effects of CEUS, and we will continue to monitor this issue.

## Conclusions

Our study demonstrated that CEUS helps to improve the knowledge of spermatic vein hemodynamics, although the feasibility of its routine use needs to be evaluated. In future studies, we will use CEUS to comprehensively and systematically explore various aspects of VC, including its natural course, surgical indications, and postoperative outcomes.

## Supplementary Information

Additional file 1. Example of the steady flow pattern.

Additional file 2. Example of the intermittent stasis pattern.

Additional file 3. Example of the intermittent reflux pattern.

Additional file 4. Example of the filling defect pattern.

## Data Availability

No datasets were generated or analysed during the current study.

## Abbreviations

VC: Varicocele
CEUS: Contrast-enhanced ultrasound
DFI: DNA fragmentation index
ICC: Intra-class correlation
FSH: Follicle-stimulating hormone
LH: Luteinizing hormone
T: Total testosterone
PRL: Prolactin
E2: Estradiol
